# Method for simultaneous analysis of eight analogues of vitamin D using liquid chromatography tandem mass spectrometry

**DOI:** 10.1186/1752-153X-6-112

**Published:** 2012-10-01

**Authors:** Iltaf Shah, Andrea Petroczi, Declan P Naughton

**Affiliations:** 1 School of Life Sciences, Kingston University, London, UK; 2 School of Life Sciences, Kingston University, Penrhyn Road, Kingston upon Thames, Surrey, KT1 2EE, UK

**Keywords:** Vitamin D, Epimer, Analyses, LC-MS/MS, 25-hydroxyvitamin-D3, 25-hydroxyvitamin-D2, 3-epi-25OHD3, 3-epi-25OHD2

## Abstract

**Background:**

Despite considerable global investigation over several decades, the roles of vitamin D in health and disease development remains convoluted. One recognised issue is the difficulty of accurately measuring the active forms of vitamin D. Advances made include some new methods addressing the potential interference by excluding epimers and isobars. However, there is no evidence that epimers are without function. Therefore, the aim of this study was to develop and validate, for the first time, a new assay to simultaneously measure levels of six forms of vitamin D along with two epimers. The assay was applied to multilevel certified reference material (CRM) and 25 pooled human sera samples, obtained from the Vitamin D External Quality Assessment Scheme (DEQAS), to demonstrate its efficiency.

**Results:**

The assay is capable of simultaneously measuring eight vitamin D analogues over the calibration ranges and LODs (in nmol/L) of: 1α25(OH)_2_D2 [0.015-1; 0.01], 1α25(OH)_2_D3 [0.1-100; 0.01], 25OHD3 [0.5-100, 0.025], 3-epi-25OHD3 [0.1-100, 0.05], 25OHD2 [0.5-100, 0.025], 3-epi-25OHD2 [0.1-100, 0.05], vitamin D3 [0.5-100, 0.05] and vitamin D2 [0.5-100, 0.05], using stanozolol-d3 as internal standard. Certified reference material and external quality control samples (DEQAS) were analysed to meet the standards outlined by National Institute of Standards and Technology (NIST). Validation steps included recovery and both precision and accuracy under inter- and intra-day variation limit of detection, and analysis of each analyte over a linear range. All validation parameters were in line with acceptable Food and Drug Administration (FDA) guidelines. All eight analogues were quantified with the 25OHD levels being commensurate with DEQAS data.

**Conclusions:**

This report details the application of a new LC-MS/MS based assay for the efficient analysis of eight analogues of vitamin D over a range of samples, which is a significant advance over the existing methods. Simultaneous measure of eight vitamin D analogues does not compromise the analytical capability of the assay to quantify the commonly used biomarker (25OHD) for vitamin D status. The results demonstrate the feasibility of applying the assay in research and clinical practice that i) excludes misleading measures owing to epimers and isobars and ii) is able to quantify the excluded component to facilitate further in vivo investigation into the roles of ubiquitous epimers.

## Background

Many studies have demonstrated that hypovitaminosis D is widespread through the Western world leading to postulations that it is associated with the development and/or progression of a number of conditions ranging from neurological disorders through to hypertension, type 1 diabetes, kidney disease and cystic fibrosis [1]. In consequence, many supplemental studies have been conducted with very few successfully altering disease outcomes [1]. Beyond rickets disease, clinical research on the putative roles of vitamin D in health and disease has proven difficult for a number of reasons including: i) the vitamin exists in multiple forms that are not facile to measure accurately, with considerable questions about several methods [2], ii) sources are varied with sunlight generating the primary form of vitamin D3 in the skin and both vitamin D3 and vitamin D2 are available through diet, making supplemental studies very difficult to control, and iii) although studies are conducted globally, there is no worldwide consensus on key quantitative measures of deficiency, insufficiency and sufficiency of vitamin D in blood samples [1,3].

Vitamin D exists in several forms ranging from the dietary derived D2 and photo-activated D3 analogues, although a fraction of vitamin D3 also arises from the diet. Hydroxylation in the liver affords the circulating 25-hydroxyvitamin-D3 (25OHD3) and 25-hydroxyvitamin-D2 (25OHD2) forms, frequently measured collectively as 25OHD. Additional metabolism in the kidney generates the active metabolites 1-alpha,25-dihydroxyvitamin-D3, (1α25(OH)_2_D3) and 1-alpha,25-dihydroxyvitamin-D2 (1α25(OH)_2_D2) along with the minor metabolite 24,25(OH)_2_D3 [4-6]. Recent efforts have resulted in significant improvements in the understanding of limitation and strengths of various assays for vitamin D forms [3,7-10].

Further confounding factors arise as recent studies have revealed the presence of the C-epimer form of 25OHD3 in adult blood samples [11], which was subsequently quantified in further studies [3,12,13]. The role of vitamin D epimers is yet to be elucidated but their presence is likely to have an associated function [14]. Given that the epimers can hamper current assays by cross-reacting, and that they may have biological functions, it is timely to develop an accurate method to analyse all forms of vitamin D, including accurate levels of the epimers. Similar efforts to date resulted in assays quantifying up to five analogues in various combinations [15-18] but none included the primary forms of vitamin D, 3-epi-25OHD2 or 1α25(OH)_2_D2.

This paucity of advances, despite the existence of numerous reports, led us to develop a new assay for the simultaneous analysis of eight key analogues of vitamin D including two epimers. The aim of this paper is to report the validated assay along with its application to reference samples to quantify the levels of various analogues of vitamin D.

## Materials and methods

### Samples

Samples were obtained from the Vitamin D External Quality Assessment Scheme (DEQAS). All the samples are pooled sera prepared from donations from patients undergoing therapeutic venesection for haemochromatosis and occasionally polycythaemia.

### Standards and reagents

All chemicals and reagents were of high purity grade. Stanozolol-D3 (internal standard) and 7-α-hydroxy-4-cholesten-3-one (7αC4) were obtained from LGC standards (Teddington, UK). Vitamin D3, vitamin D2, 25OHD3, 25OHD2, 1α25(OH)_2_D3, 1α25(OH)_2_D2 , 3-epi-25OHD3, 3-epi-25OHD2, isopropanol, dichloromethane, methanol, hexane, deionised water, formic acid, ammonium hydroxide, acetonitrile, ether and hexane were purchased from Sigma Aldrich (Poole, UK).

### Preparation of standards and samples

Methanolic stock solutions of all analytes were prepared at a concentration of 1 mg/mL and stored in amber glass vials at −80°C in the dark. All methanolic working solutions of analytes and the internal standard were prepared by serial dilution of stock solutions. A standard working solution mixture of analytes and the standalone internal standard was prepared at a concentration of 1 μg/mL.

### Sample pre-treatment

Sera samples (1 mL) were thawed equilibrated at room temperature for 15 minutes and vortex mixed. Except for the blank, 25 μL of working solution of stanozolol-d3 (internal standard) was added to all samples. 50 μL of 2 M formic acid were added, followed by 3 mL of isopropanol/methanol/ (1:1, v/v) mixture and vortex mixed to release the protein bound analyte and to promote protein precipitation during a 15 minutes incubation at 4°C. The resulting mixture was centrifuged at 3500 g for 5 minutes at 4°C to remove the suspended matter, with supernatants transferred to clean amber glass tubes for liquid-liquid extraction.

### Liquid-liquid extraction (LLE)

A 3 mL dichloromethane/hexane/(1:1, v/v) mixture was added to the remaining solution after protein precipitation and the solution was vortex mixed for 1 minute and then centrifuged at 3500 g for 5 minutes at 4°C. The upper clear layer was transferred to a new set of glass tubes with a further two extractions conducted on the residual lower layer. The extracts were pooled and dried under a gentle stream of nitrogen at room temperature prior to reconstitution in 200 μL of LC-MS grade methanol/water (1:1, v/v) solution.

### LC-MS/MS procedure

A Sciex API-3000 LC-MS/MS instrument (Applied biosystems division of MDS health group Ltd, Canada) was used for analysis together with an autosampler (PAL-CTC Analytics, Switzerland), and turbomolecular pump (1100 series, Agilent Technologies, USA). Analyst software version 1.4.2 (AB Sciex) was used for data analyses. A 10 μL aliquot of the sample was injected into the LC-MS/MS system for analysis in low light conditions. An Ultrason ES-OVM chiral column (2 mm × 150 mm, 5 μm) was used in tandem with an Agilent rapid resolution microbore Zorbax SB-C18 RRHD column (2.1 mm × 100 mm, 1.8 μm) with corresponding inlet filters for guarding the columns.

The column oven temperature was maintained at 40°C. However, the column switching programme was set in a way so that the column temperature switch from right to left after 8 minutes and then switch back to right after 13 minutes with a temperature tolerance of ±1°C. The use of switching temperatures technique resulted in good separation, better peak shape and reproducibility. Various mobile phases and differing gradient compositions were explored for optimal results with the final selected parameters as follows. Mobile phases A consisted of acetonitrile (100%) with 0.3% (v/v) formic acid and B containing 0.1% (v/v) formic acid and ammonium acetate (2 mM) with a flow rate in all steps at 0.2 mL/min. The gradient program was as follows: initial: 35% B; 0–4.0 min: a gradient change to 98% A; from 4.0–10 min: kept at 98% A; from 10–14 min: reversion of the mobile phase to 35% B; 14.1–20.0 min; 35% B.

The mass spectrometer was auto-equilibrated for 0.5 min. The channel electron multiplier (CEM) detector was set to 3290 eV with a deflection of 400. The mass spectrometer parameters like deflection potential, focussing potential, energy potential and collision cell exit potential was optimised for each analyte. The mass spectrometer was set to run three periods sequentially with optimised conditions for selected analytes. The total run time for mass spectrometer was 20 minutes. First period was run for 8 minutes with optimum conditions for 1α25(OH)_2_D3 and 1α25(OH)_2_D2 and stanozolol-d3 internal standard followed by second period for 5 minutes with optimised conditions for 25OHD3, 25OHD2, 3-epi-25OHD3, 3-epi-25OHD2 and 7αC4 and a 3^rd^ period was employed for a final 7 minutes for determination of vitamin D3 and vitamin D2. The mass spectrometer was operated in positive electrospray ionisation (ESI) mode at an average spray voltage of 4500 V and average capillary temperature of 450°C between the three periods. The generated protonated molecules of all analytes were used as precursor ions for collision activated dissociation (CAD) into product ions in MS-MS analysis.

### Data analysis

Results from the new assay was compared to the DEQAS mean using Bland–Altman plot to allow showing discrepancies for individual samples. Coefficient of variation (CV) was calculated as standard deviation/mean and expressed as percentage. Data analysis was performed using Microsoft Excel 2007.

## Results and discussion

### Method validation

**Figure 1:**
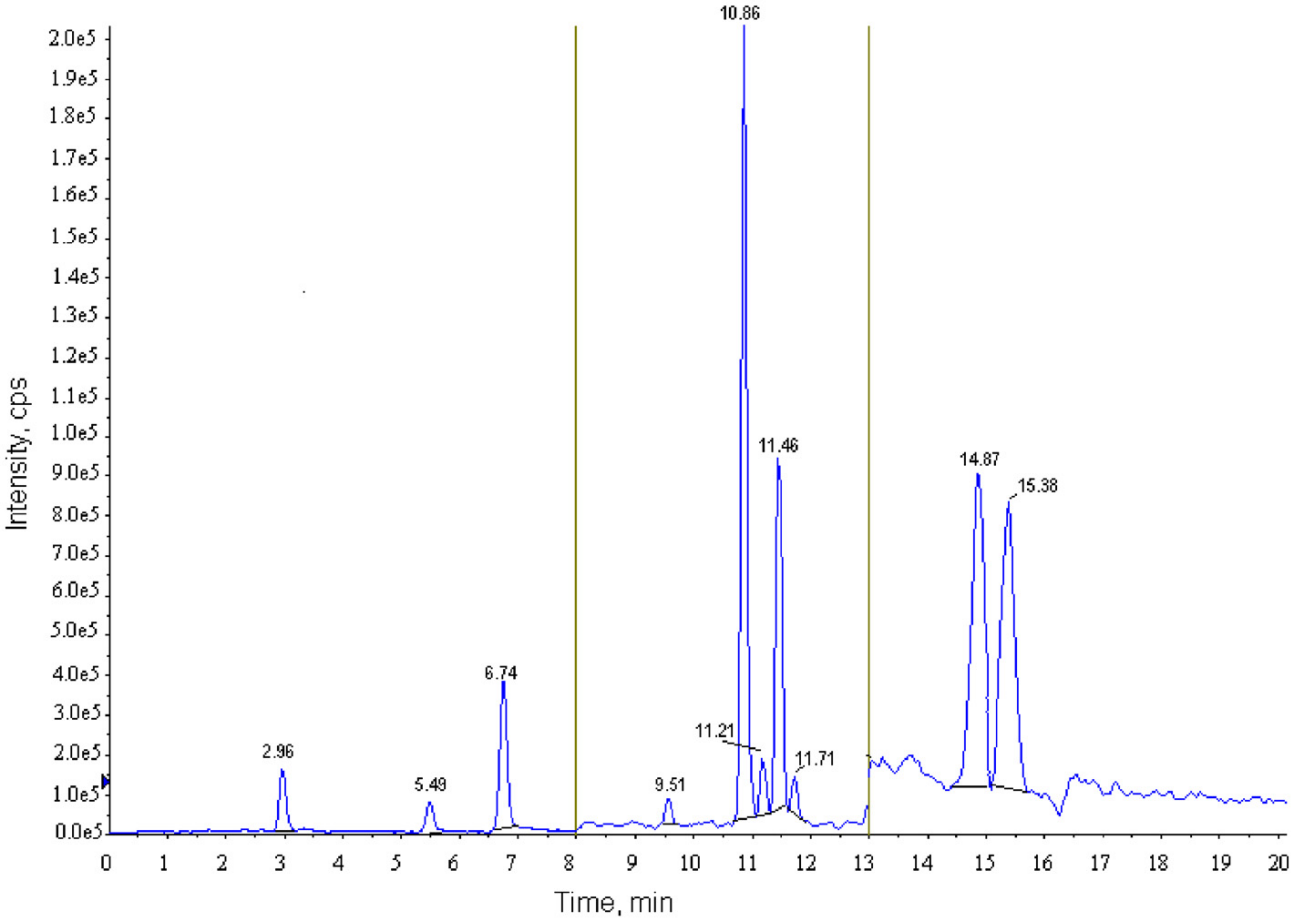
**Typical chromatogram showing vitamin D analogues [1**^**ST **^**period analytes: Stanozolol-d3-RT = 2.96, 1α25(OH)**_**2**_**D2-RT = 5.49, 1α25(OH)**_**2**_**D3-RT = 6.74; 2**^**nd **^**period analytes: 7αC4-RT = 9.51, 25OHD3-RT = 10.86, 3-epi-25OHD3-RT = 11.21, 25OHD2-RT = 11.46 and 3-epi-25OHD2-RT = 11.71; 3**^**RD **^**period analytes: vitamin D3-RT = 14.87 and vitamin D2-RT = 15.38].**

A typical chromatogram is shown in Figure 1 illustrating the separation of eight vitamin D analogues plus the internal standard (stanozolol-d3) and isobaric component 7-α-hydroxy-4-cholesten-3-one (7αC4) in a spiked extracted serum sample. The calibration curves and quality controls were prepared in vitamin d free phosphate-buffered saline with 60 g/L human serum albumin [19]. The sample pre-treatment and extraction method was followed with minor changes [19]. The accuracy of the assay was determined as the ratio of the compound added to the measured (mean value/nominal value) × 100. The extraction efficiency of the compounds was determined by spiking blank serum samples prepared in human serum albumin. Absolute recoveries were determined by comparing the concentration of the detector response obtained from peak heights ratios of extracted serum samples with actually added concentrations of un-extracted standard response for the detector. Relative recoveries were determined by comparing the % of drug recovered from serum matrix against detector response for extracted standards. Three different concentrations of quality controls were utilised in this assay.

To check for the assay variations for 25OHD3 and 25OHD2, a multilevel certified reference material (CRM) serum calibrator set (Chromsystems, Germany) was also used (based on human serum) and handled in the same manner as volunteer specimens [3]. The CRM calibrators were analysed along with routine samples to meet the standards outlined by National Institute of Standards and Technology (NIST). External quality control samples from the International Vitamin D External Quality Assessment Scheme (DEQAS) were analysed for comparison with other participating laboratories already using LC-MS/MS for 25OHD3 and 25OHD2 analysis.

### Validation results

Intra-day and inter-day precision and accuracy are shown in Table 1. The absolute recoveries of the analytes range from 88.2 to 97.8% and internal standard 89.6%. Relative recoveries for analytes ranged from 89.8 to 98.2% and internal standard 89.9%. The r^2^ in Table 1 represents linear regression, where all the results are presented in units of nmol/L.

**Table 1:** **Validation results for vitaminD assay**

**Analytes in(nmol**/**L)**	**Conctrn.QC's**	**Linear range**	**LOD**	**r**^**2**^	**Intra**-**day**		**Inter**-**day**	
					**Precision % CV**	**Accuracy%**	**Precision % CV**	**Accuracy%**
Vitamin D3	20	0.5-100	0.05	0.9997	4.13	87.1	5.32	89.8
	40				2.86	99.4	2.33	89.4
	80				1.31	98.9	2.89	90.2
Vitamin D2	20	0.5-100	0.05	0.9996	6.3	99.2	11.3	93.2
	40				11.3	97.5	13.3	94.8
	80				9.4	89.4	9.6	89.3
25OHD3	20	0.5-100	0.025	0.9999	6.1	99.9	7.4	98.1
	40				2.7	97.3	3.7	99.8
	80				3.5	99.1	2.9	99.2
25OHD2	20	0.5-100	0.025	0.9998	2.8	99.8	3.4	101.2
	40				2.7	99.9	3.9	98.9
	80				3.3	88.7	3.8	89.3
1α25(OH)_2_ D3	0.2	0.015-1	0.010	0.9998	4.4	89.2	5.6	88.5
	0.4				4.2	86.4	4.4	87.3
	0.8				3.6	99.7	2.6	88.9
1α25(OH)_2_ D2	0.2	0.015-1	0.010	0.9998	3.2	98.9	5.2	89.8
	0.4				4.4	99.4	6.3	98.9
	0.8				3.9	99.9	4.3	87.8
3-epi-25OHD3	20	0.1-100	0.05	0.9997	3.5	89.2	4.3	98.5
	40				3.1	87.3	10.9	87.6
	80				3.3	89.1	8.9	99.9
3-epi-25OHD2	20	0.1-100	0.05	0.9998	7.1	85.8	3.5	88.5
	40				4.9	84.6	2.7	87.1
	80				3.2	89.9	2.2	88.3
7αC4	20	0.5-100	0.1	0.9999	3.2	98.3	5.4	86.2
	40				2.3	88.6	3.1	85.3
	80				4.1	79.9	3.1	88.1

Under- or over-estimation of actual 25OHD3 concentrations may occur owing to co-eluting epimers (e.g. 3-epi-25OHD3) and isobars (e.g. 7αC4). The endogenous bile acid precursor 7α-hydroxy-4-cholestene-3-one (7αC4) may cause isobaric inference in MS assays [3]. The use of high resolution microbore tandem column technology not only chromatographically separated all isobars and epimers from 25OHD co-eluting peaks but also facilitated accurate and sensitive determination of the analyte ions by sequential application of three periods during an MS/MS run. The protonated molecules, [M + H]^+^, the precursor ions and the diagnostic product ions were monitored in the multiple reactions monitoring (MRM) mode. The MRM parameters were optimised for each analyte using a direct infusion of 0.1 μg/mL individual solutions and of standard compounds mixture. The MRM sequence consisted of three periods executed sequentially to monitor different transition pairs using parameters optimised for each period. The most abundant MRM transitions for each analyte were acquired using the conditions given in Table 2.

**Table 2:** **MRM transitions of vitaminD analogues and internalstandard**

**Compounds**	**Precursor**	**Product**	**CE**
Vitamin D3	385.3	159.2	40
Vitamin D2	397.4	159.2	43
25OHD3	401.3	383.1	41
	401.3	365.1	41
	401.3	159.2	45
25OHD2	413.3	377.2	51
	413.3	355.5	51
	413.3	383.1	35
1α25(OH)_2_ D3	399.1	135.1	61
	399.1	381.1	41
1α25(OH)_2_ D2	411.5	151.1	55
3-epi-25OHD3	401.3	383.1	41
	401.3	365.1	41
	401.3	159.2	45
3-epi-25OHD2	413.3	377.2	51
	413.3	355.5	51
	413.3	383.1	35
7αC4	401.3	383.1	41
	401.3	365.1	41
	401.3	159.2	45
Stanozolol-D3	332.2	81.2	45

### Profiles of vitamin D analogues

**Figure 2:**
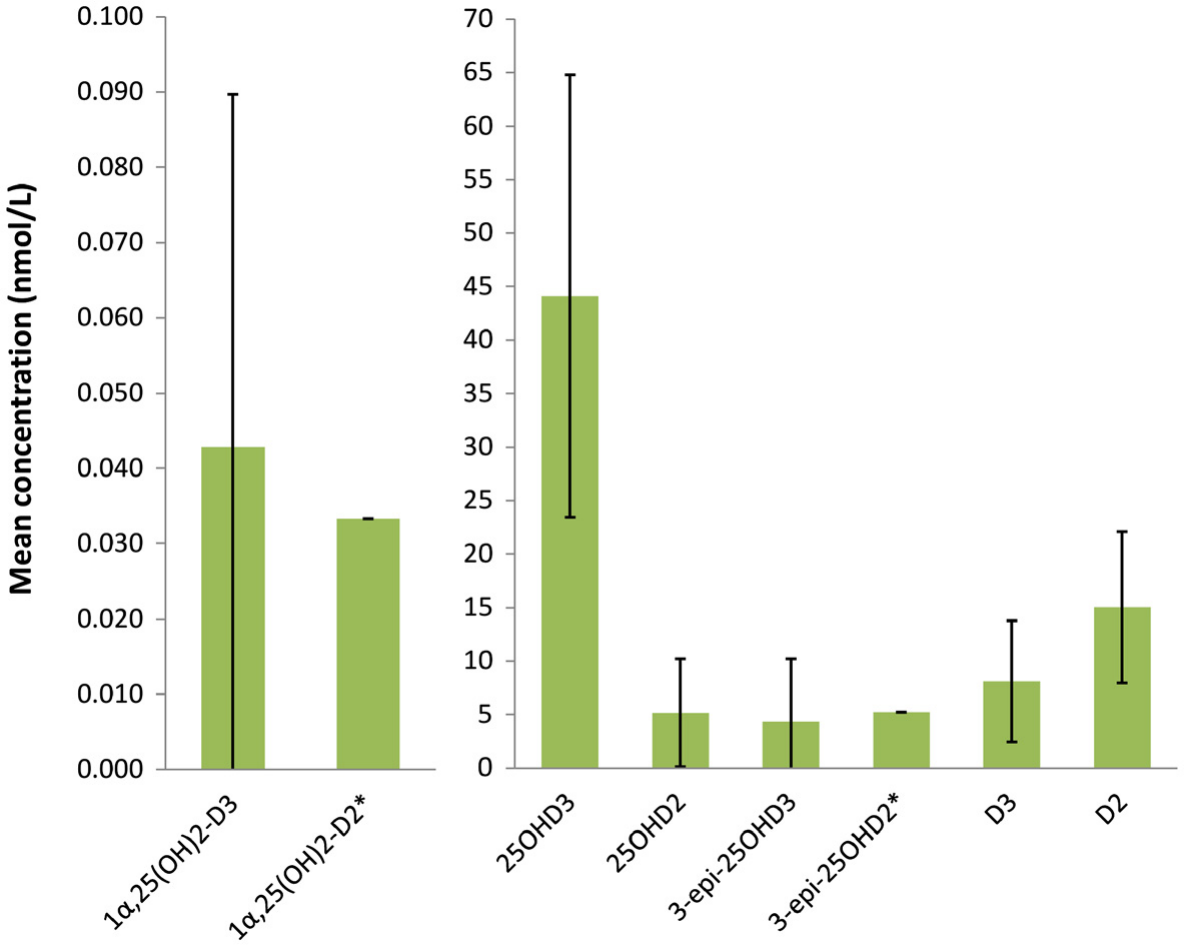
**Analytical data for DEQAS samples using the new assay.** * indicates that single value is presented as no mean value is available only one positive sample was recorded. The error bars represent standard deviations for the 25 samples.

The results from the pooled DEQAS samples are given in Figure 2 revealing that all analogues were quantifiable. As expected, the highest level was recorded for 25OHD3 with 25OHD2 present at a considerable level in all samples. The C-3 epimer of 25OHD3 and 1α25(OH)_2_D2 were quantified in most samples, whereas the 1α25(OH)_2_D2 and 3-epi-25OHD2 were only present in one sample (410), which contained a significant amount of endogenous 25OHD2. It should be noted, however, that these are pooled samples from donors undergoing treatment and thus are used for the purposes of assay comparisons.

**Figure 3:**
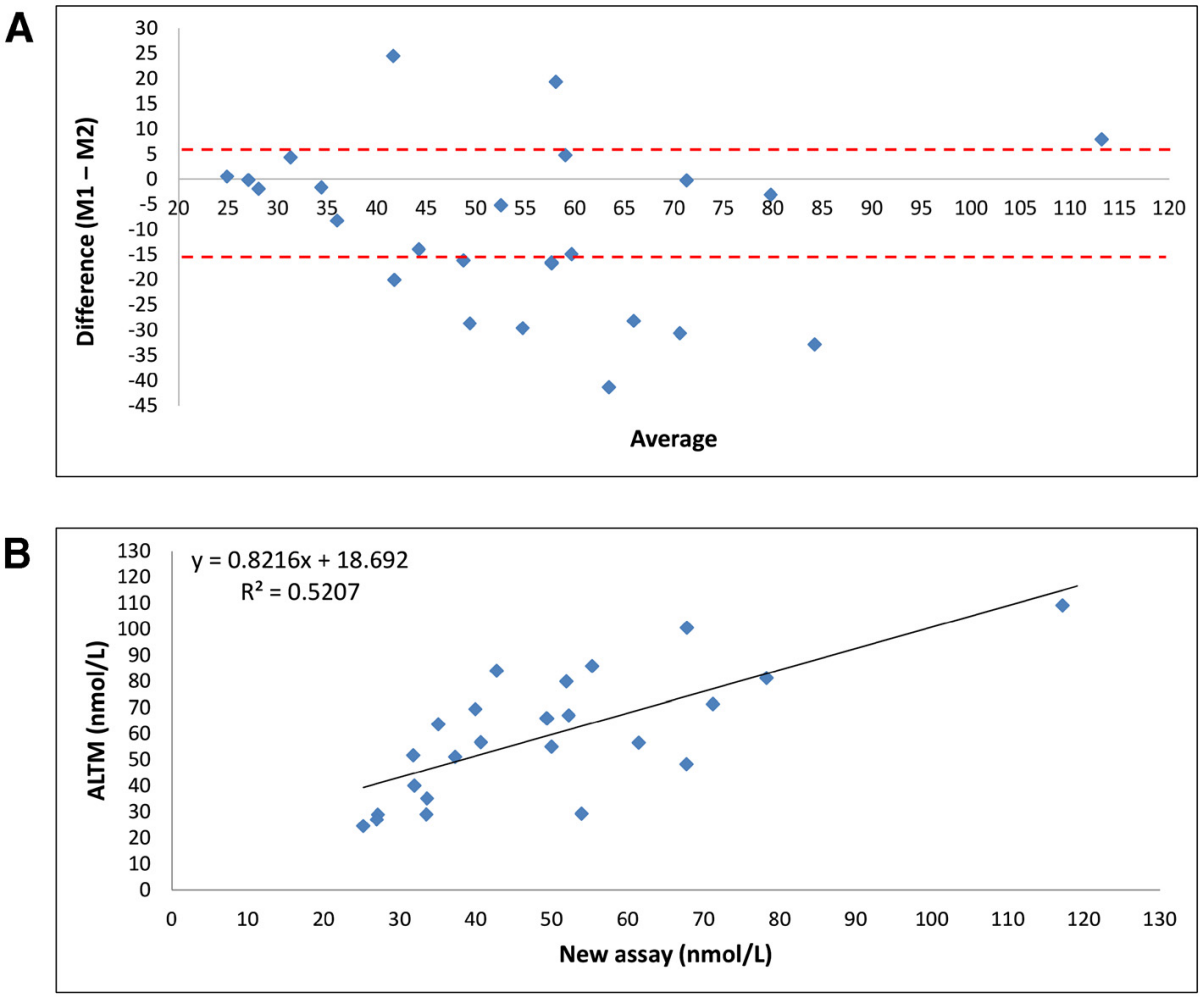
**A: Bland-Altman plot comparing the 25OHD concentrations obtained withthe new assay (M1) to DEQAS.** Lines represent limits of agreement. The mean difference is −5.329, the lower limit line is at −15.774 and the upper limit is at 5.116. B: Correlation between the 25OHD concentrations obtained using the new assay and the ALTM.

**Figure 4:**
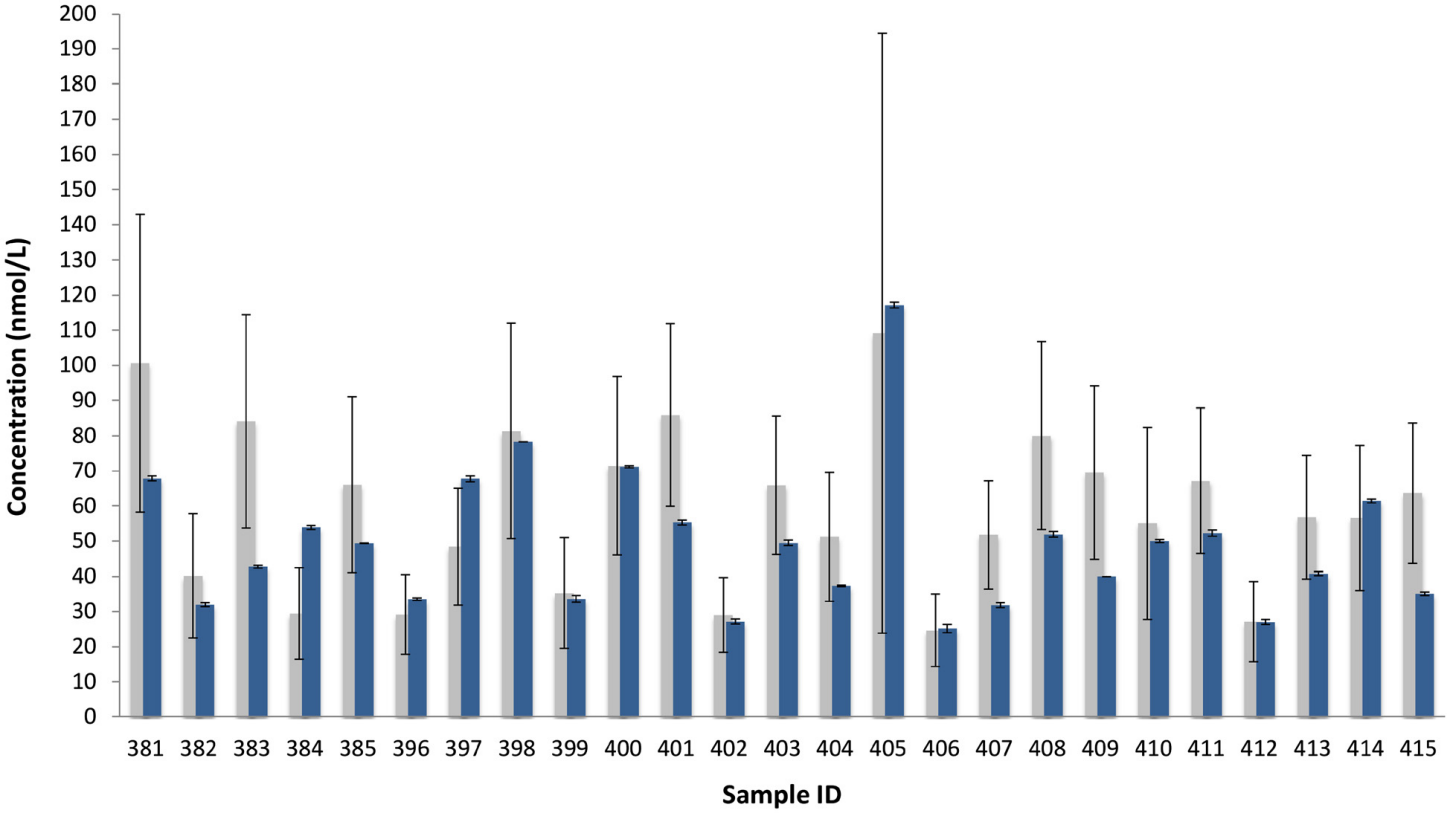
**Comparison of 25OHD concentration levels obtained using the new assay (blue) and ALTM (grey).** Bars for new assay represent SEM of duplicate measures. Bars on ALTM represent CV = 30.0 and denote the lower and upper limit of satisfactory performance [ http://www.deqas.org].

The Bland-Altman and correlation plots (Figure 3) show the level of agreement and relationship between the new method and the All Laboratory Trimmed Mean (ALTM) data provided with the DEQAS sample. The analytical data 25OHD for the individual samples are presented in Figure 4 with detailed results given in Additional file 1. As the Bland-Altman plot showed no proportional bias (r^2^ = 0.0341, p = 0.376), the classic 96% confidence interval approach was applied using the Bland-Altman formula (mean difference ± 1.96SD) to set the limits of agreement. Of the total 25 samples, 15 were within the limits of agreement by Bland-Altman limit of agreement (Figure 3A); and 18 were within the DEQAS CV = 30.0 limit (Figure 4). Generally, the 25OHD concentration level measures falling outside the Bland-Altman and DEQAS limits were recorded at a lower level than the DEQAS’ ALTM means and confidence intervals. A strong positive relationship was evidenced between the new assay and the ALTM (r = 0.72, p < 0.001). Our data were within the limit of tolerance of the subset of 5 pooled DEQAS samples (for samples 396–400) which were analysed by the Centers for Disease Control and Prevention (Atlanta) with known 3-epi-25OHD3 levels.

Our results for the serum pools averaged −11.8% from the overall DEQAS-ALTM for the participating laboratories using LC-MS/MS methods. However, we did see a diversification in some samples that afforded higher concentrations of the 25OHD. Thus, as seen in Figure 4, five results for the new assay lie below the range of the DEQAS data (383, 401, 407, 409, 415), with two samples being above the DEQAS (384, 397).

## Conclusion

In conjunction with the ability to separate 7αC4, the assay is capable of distinguishing between every major form of vitamin D reported to date. The results show that the new assay’s ability to quantify 25OHD levels is comparable to other LC-MS/MS methods represented in the DEQAS data, thus the simultaneous measures of eight analogues does not compromise its analytical capability for assessing the commonly used biomarker (25OHD) for vitamin D status. In contrast, it adds a significant capability to identify potential issues such as conversion of primary forms to circulating or highly active forms [19]. However, the additional capability of the new assay, namely the ability of not only excluding but also quantifying 3-epimers of D2 and D3, along with the primary forms (D2 and D3), 1,25α(OH)_2_D3 and 1,25α(OH)_2_D2, makes this assay instrumental in research and clinical practice where specific and accurate measurement of the different forms is required.

## Competing interests

The authors declare that they have no conflicts of interest.

## Authors’ contributions

DPN and AP initiated the study. The method development, validation and sample analyses were conducted by IS with contributions from DPN. AP conducted the data analyses. All authors contributed to the study design, interpretation of the results and preparation of the manuscript and have read and approved the final version.

## Supplementary Material

Additional file 1 **The following additional data are available with the online version of this paper.** Additional data file 1 is a table listing the concentrations ± SEM of the 8 vitamin D forms (nmol/L) in DEQAS pooled samples. Click here for file

## References

[B1] Zhang R Naughton DP Vitamin D in health and disease: current perspectives Nutr J 2010 9 65 21143872 10.1186/1475-2891-9-65 PMC3019131

[B2] Carter GD 25-Hydroxyvitamin D: a difficult analyte Clin Chem 2012 58 3 486 488 22238254 10.1373/clinchem.2011.180562

[B3] Shah I James R Barker J Petroczi A Naughton DP Misleading measures in vitamin D analysis: a novel LC–MS/MS assay to account for epimers and isobars Nutr J 2011 10 46 21569549 10.1186/1475-2891-10-46 PMC3114718

[B4] Hollis BW Circulating 25-hydroxyvitamin D levels indicative of Vitamin D sufficiency: implications for establishing a new effective dietary intake recommendation for vitamin D J Nutr 2005 135 317 322 15671234 10.1093/jn/135.2.317

[B5] Barragry JM France MW Corless D Gupta SP Switala S Boucher BJ Cohen RD Intestinal cholecalciferol absorption in the elderly and in younger adults Clin Sci Mol Med 1978 55 213 220 209929 10.1042/cs0550213

[B6] Barragry JM France MW Boucher BJ Cohen RD Metabolism of intravenously administered cholecalciferol in man Clin Endocrinol (Oxf) 1979 11 491 495 93036 10.1111/j.1365-2265.1979.tb03101.x

[B7] Saenger AK Laha TJ Bremner DE Sadrzadeh SMH Quantification of serum 25-hydroxyvitamin D(2) and D(3) using HPLC-tandem mass spectrometry and examination of reference intervals for diagnosis of vitamin D deficiency Am J Clin Pathol 2006 125 914 920 16690491 10.1309/J32U-F7GT-QPWN-25AP

[B8] Couchman L Benton CM Moniz CF Variability in the analysis of 25-hydroxyvitamin D by liquid chromatography-tandem mass spectrometry: the devil is in the detail Clin Chim Acta 2012 413 1239 1243 22515958 10.1016/j.cca.2012.04.003

[B9] Wallace AM Gibson S de la Hunty A Lamberg-Allardt C Ashwell M Measurement of 25-hydroxyvitamin D in the clinical laboratory: Current procedures, performance characteristics and limitations Steroids 2010 75 477 488 20188118 10.1016/j.steroids.2010.02.012

[B10] Janssen MJW Wielders JPM Bekker CC Boesten LSM Buijs MM Heijboer AC Van Der Horst FAL Loupatty FJ Van Den Ouweland JMW Multicenter comparison study of current methods to measure 25-hydroxyvitamin D in serum Nederlands Tijdschrift voor Klinische Chemie en Laboratoriumgeneeskunde 2012 37 223 226

[B11] Singh RJ Taylor RL Reddy GS Grebe SK C-3 epimers can account for a significant proportion of total circulating 25-hydroxyvitamin D in infants, complicating accurate measurement and interpretation of vitamin D status J Clin Endocrinol Metab 2006 91 3055 3061 16720650 10.1210/jc.2006-0710

[B12] Lensmeyer G Poquette M Wiebe D Binkley N The C-3 epimer of 25-hydroxyvitamin D(3) is present in adult serum J Clin Endocrinol Metab 2012 97 163 168 22013102 10.1210/jc.2011-0584

[B13] Strathmann FG Sadilkova K Laha TJ LeSourd SE Bornhorst JA Hoofnagle AN Jack R 3-epi-25 hydroxyvitamin D concentrations are not correlated with age in a cohort of infants and adults Clin Chim Acta 2012 413 203 206 21983164 10.1016/j.cca.2011.09.028 PMC3236660

[B14] Jones G Metabolism and biomarkers of Vitamin D Scand J Clin Lab Investigation 2012 S243 7 13 10.3109/00365513.2012.681892 22536757

[B15] Schleicher RL Encisco SE Chaudhary-Webb M Paliakov E McCoy LF Pfeiffer CM Isotope dilution ultra performance liquid chromatography-tandem mass spectrometry method for simultaneous measurement of 25-hydroxyvitamin D2, 25-hydroxyvitamin D3 and 3-epi-25-hydroxyvitamin D3 in human serum Clin Chim Acta 2011 412 1594 1599 21601563 10.1016/j.cca.2011.05.010

[B16] Baecher S Leinenbach A Wright JA Pongratz S Kobold U Thiele R Simultaneous quantification of four vitamin D metabolites in human serum using high performance liquid chromatography tandem mass spectrometry for vitamin D profiling Clin Biochem 2012 10.1016/j.clinbiochem.2012.06.030 22771503

[B17] Ding S Schoenmakers I Jones K Koulman A Prentice A Volmer DA Quantitative determination of vitamin D metabolites in plasma using UHPLC-MS/MS Anal Bioanal Chem 2010 398 779 789 20628873 10.1007/s00216-010-3993-0 PMC2939348

[B18] Wang Z Senn T Kalhorn T Zheng XE Zheng S Davis CL Hebert MF Lin YS Thummel KE Simultaneous measurement of plasma vitamin D(3) metabolites, including 4β,25-dihydroxyvitamin D(3), using liquid chromatography-tandem mass spectrometry Anal Biochem 2011 418 126 133 21784054 10.1016/j.ab.2011.06.043 PMC3164754

[B19] Shah I Petroczi A Tabet N Klugman A Isaac M Naughton DP Acetylcholine esterase inhibitors normalise circulating vitamin D2 levels in Alzheimer’s disease: an observational study Curr Alzheimer Res 2012 in press 10.2174/156720512803568975 22876849

