# Supplementary material for: Method for simultaneous analysis of eight analogues of vitamin D using liquid chromatography tandem mass spectrometry
Source: Chem Cent J. 2012 Oct 1;6:112. doi: 10.1186/1752-153X-6-112 (PMC3505479; doi:10.1186/1752-153X-6-112)
Supplement: Additional file 1 — The following additional data are available with the online version of this paper. Additional data file 1 is a table listing the concentrations ± SEM of the 8 vitamin D forms (nmol/L) in DEQAS pooled samples. [file 1752-153X-6-112-S1.pdf]

Concentrations ± SEM of the 8 vitamin D forms (nmol/L) in DEQAS pooled samples. Measures were taken in duplicates.

ID	1α,25$(OH)_2$-D3	1α,25$(OH)_2$-D2	25-OH-D3	25-OH-D2	3-epi-25OHD3	3-epi-25OHD2	V-D3	V-D2
**381**	0.024±0.00313	0.000	64.72±0.41150	3.073±0.04750	4.713±0.00250	0.000	8.845±0.00500	6.910±0.02000
**382**	0.021±0.00045	0.000	26.570±0.33000	5.338±0.10750	3.538±0.04825	0.000	12.745±0.51500	8.218±0.09800
**383**	0.022±0.00079	0.000	38.965±0.24525	3.792±0.20150	8.500±0.05025	0.000	11.340±0.36000	7.196±0.01400
**384**	0.031±0.00028	0.000	48.793±0.32700	5.142±0.18200	3.775±0.02475	0.000	12.300±0.18000	21.707±0.41300
**385**	0.022±0.00047	0.000	46.068±0.04250	3.239±0.02850	0.000	0.000	11.710±0.27000	7.083±0.02700
**396**	0.021±0.00083	0.000	32.175±0.19500	1.335±0.00500	1.370±0.02000	0.000	0.000	12.226±0.00400
**397**	0.021±0.00043	0.000	66.625±0.49500	1.120±0.01000	2.225±0.00500	0.000	0.000	21.616±0.05600
**398**	0.023±0.00156	0.000	76.941±0.00050	1.300±0.01000	3.960±0.02000	0.000	8.680±0.03000	28.280±0.07000
**399**	0.021±0.00013	0.000	32.603±0.60676	0.995±0.01500	3.115±0.00500	0.000	9.230±0.00000	22.392±0.16200
**400**	0.026±0.00500	0.000	69.735±0.17500	1.455±0.06500	2.955±0.00500	0.000	6.625±0.00500	30.180±0.06000
**401**	0.031±0.00005	0.000	52.301±0.41125	3.018±0.00750	2.264±0.01418	0.000	6.245±0.12500	29.285±0.32500
**402**	0.022±0.00061	0.000	26.707±0.41300	0.420±0.01000	2.158±0.00815	0.000	6.699±0.04850	12.289±0.05900
**403**	0.020±0.00001	0.000	43.858±0.45200	5.543±0.02300	6.916±0.00610	0.000	5.657±0.03700	13.364±0.24400
**404**	0.051±0.00021	0.000	33.016±0.10375	4.273±0.03750	1.553±0.00313	0.000	0.000	9.834±0.00600
**405**	0.083±0.00140	0.000	111.629±0.49075	5.548±0.01800	29.010±0.11000	0.000	0.000	11.458±0.00800
**406**	0.022±0.00215	0.000	15.043±0.70251	10.142±0.02200	3.439±0.00933	0.000	14.125±0.08500	13.294±0.31400
**407**	0.031±0.00037	0.000	28.735±0.38475	3.028±0.00250	4.354±0.00375	0.000	19.820±0.17000	11.977±0.13300
**408**	0.022±0.00098	0.000	43.045±0.48525	8.863±0.01300	1.126±0.00648	0.000	11.540±0.31000	13.023±0.04310
**409**	0.031±0.00051	0.000	37.847±0.00325	2.105±0.00500	3.948±0.00168	0.000	19.570±0.45000	11.619±0.38890
**410**	0.021±0.00026	0.033±0.00081	29.876±0.24450	20.094±0.11350	1.114±0.00598	5.215±0.01500	2.985±0.00500	11.659±0.00880
**411**	0.116±0.00466	0.000	43.596±0.52376	8.626±0.08600	0.976±0.00445	0.000	0.000	11.356±0.23600
**412**	0.107±0.00608	0.000	26.860±0.41000	0.125±0.00500	1.483±0.02323	0.000	5.680±0.04000	10.926±0.03600
**413**	0.031±0.00026	0.000	34.299±0.31948	6.382±0.03150	0.927±0.00688	0.000	6.700±0.06000	11.930±0.04000
**414**	0.228±0.01657	0.000	42.705±0.34450	18.749±0.24850	13.459±0.10148	0.000	9.785±0.09500	23.500±0.05000
**415**	0.022±0.00211	0.000	29.867±0.30675	5.225±0.01500	1.165±0.04500	0.000	11.780±0.22000	14.065±0.04500
